# Caught Between Care and Collapse: An Interpretive Qualitative Exploration of Burnout and Resilience Among Respiratory Therapists in Saudi Arabia

**DOI:** 10.3390/healthcare14040504

**Published:** 2026-02-15

**Authors:** Rayan A. Siraj, Maryam M. Almulhem

**Affiliations:** Department of Respiratory Therapy, College of Applied Medical Sciences, King Faisal University, Al-Ahsa 31982, Saudi Arabia; mmalmulhim@kfu.edu.sa

**Keywords:** burnout, respiratory therapists, coping, organizational support, Saudi Arabia

## Abstract

Background: Although burnout among respiratory therapists (RTs) is well documented, qualitative insights into their lived experiences in Saudi Arabia remain limited. This study explored RTs’ experiences of burnout, systemic and organisational drivers of professional strain, and strategies for resilience and retention within Saudi hospitals. Methods: A qualitative descriptive design was employed. Purposive sampling was used to recruit 11 RTs from diverse regions across Saudi Arabia. Semi-structured interviews were conducted in Arabic between September and November 2025, audio-recorded, and transcribed verbatim. Data management and analysis followed a hybrid approach using NVivo 12 software alongside manual coding to support deep immersion in the data. Analysis was guided by Braun and Clarke’s reflexive thematic analysis. Methodological rigour was enhanced through reflexive memoing, peer debriefing, and adherence to a 15-point trustworthiness checklist. Results: Analysis generated one overarching theme, “Caught Between Care and Collapse: The Human Cost of Institutional Burnout,” alongside three interrelated themes. Participants described (1) “Living within a system that drains the self,” highlighting sustained physical and emotional exhaustion driven by understaffing and extended shifts; (2) “Losing meaning and recognition,” illustrating how organisational neglect eroded professional passion and replaced it with obligation and frustration; and (3) “Coping strategies and informal support,” reflecting quiet resilience through self-regulation, peer solidarity, and humane leadership. Many participants framed their endurance as an act of moral defiance rather than passive resignation. Conclusions: These findings suggest that RT burnout reflects not individual failure but a structural outcome of sustained strain and deficits in reciprocity. Burnout emerges as an institutional crisis in which therapists remain deeply committed to patient care while being pushed toward professional collapse by systemic neglect. Culturally informed, system-level interventions are urgently needed to preserve this essential workforce.

## 1. Introduction

Burnout is a prolonged response to chronic emotional and interpersonal stressors at work and is defined by three core dimensions, namely exhaustion, cynicism, and inefficacy, commonly operationalised as emotional exhaustion (EE), depersonalisation (DP), and a reduced sense of personal accomplishment (PA) [1]. Consistent with this conceptualisation, the World Health Organisation (WHO) classifies burnout in the International Classification of Diseases, Eleventh Revision (ICD-11) as an occupational phenomenon rather than a medical condition, emphasising its relationship to chronic workplace stress. Among healthcare professionals (HCPs) working in high-stress environments such as intensive care units (ICUs) and emergency departments (ERs), burnout is associated with impaired well-being [2], poorer clinical performance [3], increased medical errors [4], and compromised patient safety [5].

Although respiratory therapists (RTs) are crucial frontline clinicians in managing acute and chronic respiratory failure, particularly in critical care settings, they are nevertheless an understudied group in burnout research. In Saudi Arabia, quantitative studies indicate a high prevalence of burnout. A previous hospital-based cross-sectional study found that 77% of RTs had emotional exhaustion, 98% reported depersonalisation, and 73% felt low personal accomplishment [6]. A national analysis further identified long work hours, poor supervisory support, and low resilience as key contributors to an increased risk of burnout [7]. While these findings establish the magnitude of the problem, they offer limited insight into how burnout is subjectively experienced, interpreted, and navigated by RTs in everyday clinical practice.

There is evidence to suggest that burnout is strongly influenced by the context in which HCPs work. Indeed, a large Saudi comparative study of 844 healthcare workers noted that ICU staff, especially RTs and nurses, showed significantly higher EE and DP and lower PA than non-ICU staff [8]. The latter study indicated that sociodemographic factors, including marital status, experience, shift length, and secondary employment, significantly influenced severity. Within the Saudi healthcare system, hierarchical organisational structures, workforce nationalisation policies, and reliance on shift-based critical care staffing may further shape how burnout is experienced and expressed by RTs.

Despite this data, qualitative insights into RTs’ lived burnout experiences are currently scarce. A prior qualitative study of ICU clinicians, which included only three RTs, found that non-physician staff often felt undervalued [9]. Further research in Qatar indicated that perceived leadership and empowerment significantly affect RT burnout, highlighting the role of interpersonal and organisational context [10]. Notably, although burnout and mental health research among healthcare professionals has expanded substantially in recent years, particularly following the COVID-19 pandemic, recent qualitative studies focusing specifically on respiratory therapists remain limited, suggesting a stagnation in profession-centred research rather than a lack of relevance.

Addressing burnout requires a two-pronged approach combining systemic and individual strategies. Organisational interventions—such as workload management, psychological support, flexible schedules, and career development—can improve staff well-being [9]. Individually, coping mechanisms such as mindfulness, exercise, and peer support are common, though their efficacy depends on personal resilience, workplace culture, and institutional support. Nevertheless, how Saudi RTs personally cope with burnout and how they view organisational support remains to be ascertained.

Beyond its impact on psychological health and job satisfaction, burnout is also associated with increased intentions to leave the profession. Quantitative data from Saudi Arabia confirm that high burnout scores and limited supervisory support are associated with a stronger intent to leave the profession [7]. However, the subjective processes by which RTs interpret burnout and make decisions about professional retention are not well documented.

Overall, the existing literature in Saudi Arabia is predominantly cross-sectional and quantitative, limiting understanding of the subjective, relational, and contextual dimensions of burnout. Quantitative approaches are inherently constrained in their ability to capture meaning-making processes, identity negotiations, and lived experiences within complex organisational settings. Adopting an interpretive qualitative approach, the present study seeks to understand burnout as a socially situated and context-dependent phenomenon rather than solely a measurable outcome.

Accordingly, this study was guided by the following research questions:

(1) How do respiratory therapists in Saudi Arabia describe their lived experiences of burnout?
(2) What workplace and organisational factors do they perceive as contributing to burnout?
(3) How do respiratory therapists experience and navigate resilience and decisions related to professional retention?”

## 2. Methods

### 2.1. Study Design and Setting

A qualitative descriptive design was used to examine RTs’ experiences of workplace burnout. This approach was chosen to produce detailed, practice-oriented accounts that remain close to participants’ own descriptions and meanings. Within a pragmatic interpretive epistemology, qualitative description was considered appropriate for examining how burnout is experienced and understood in routine clinical settings, without aiming to generate theory or engage in phenomenological abstraction. Semi-structured interviews were conducted across multiple government hospitals in Saudi Arabia, including intensive care units (ICUs), emergency departments (ERs), and general wards, to capture variation in clinical environments and organisational contexts.

### 2.2. Participants and Sampling

Participants were licensed respiratory therapists actively working in Saudi Arabia, all with at least two years of clinical experience to ensure familiarity with workplace routines and occupational stressors. A purposive sampling technique was used to achieve variation across individual and structural characteristics, including age, gender, years of experience, clinical setting, and geographic region. Recruitment occurred through professional networks, hospital departments, and online platforms, enabling the inclusion of respiratory therapists from multiple regions of Saudi Arabia.

To minimise potential coercion or perceived obligation, participation was entirely voluntary, recruitment messages explicitly emphasised independence from managerial oversight, and interviews were arranged outside formal reporting structures where possible. These measures were intended to support open participation and reduce the influence of institutional dynamics on participants’ responses.

While the sample was purposively varied across demographic and organisational characteristics, it was considered sufficiently homogeneous in terms of professional identity and experiential focus. All participants were respiratory therapists operating within comparable healthcare systems and organisational environments and were exposed to similar occupational stressors inherent to respiratory care practice. This balance between structural heterogeneity and experiential homogeneity was considered appropriate to support in-depth qualitative exploration of burnout experiences.

The sample included respiratory therapists working in frontline clinical roles as well as those holding supervisory or leadership positions. Importantly, leadership roles in the participating settings did not exclude continued frontline clinical responsibilities; all participants, including those in supervisory positions, remained regularly engaged in direct patient care and were therefore exposed to comparable levels of clinical workload and occupational stress. Professional role was nevertheless treated as a relevant sampling characteristic, as leadership responsibilities may shape perceptions of organisational support, accountability, and decision-making alongside frontline practice.

Participants were recruited from multiple regions within Saudi Arabia, although regional representation was uneven, with a higher proportion from the Western region. The study prioritised depth of experiential insight rather than geographic representativeness; regional location was therefore treated as a contextual feature rather than a comparative analytic variable.

Eleven participants were interviewed, and their characteristics are summarised in Table 1. Sampling adequacy was guided by the concept of information power [9], which considers the richness and relevance of data in relation to the study aims. Information power was assessed iteratively during data analysis, based on the richness of participant narratives, recurrence of experiential patterns across interviews, and the emergence of conceptually coherent themes related to burnout, organisational context, and professional identity. Sample adequacy was therefore determined by analytic density and thematic coherence rather than numerical sufficiency alone.

Variation in participants’ years of experience, gender, clinical roles, and settings was analytically leveraged to examine convergence and divergence in burnout experiences, rather than to support demographic comparison.

The research team, composed of academically trained respiratory therapists, facilitated participant access and rapport-building. Reflexive notes were maintained throughout recruitment and data collection to support transparency and to critically reflect on potential influences related to professional proximity and insider positioning.

### 2.3. Data Collection

Data were collected through one-on-one semi-structured interviews conducted between 1 September and 15 November 2025. To accommodate participants’ preferences and geographic locations, interviews were carried out either in person or online via secure video conferencing software such as Zoom. This flexible approach enabled broader inclusion of respiratory therapists from different regions and healthcare facilities across Saudi Arabia.

The use of both in-person and online interview modalities was considered reflexively during data collection and analysis. In-person interviews allowed for greater observation of nonverbal cues and workplace context, whereas online interviews afforded participants increased privacy and scheduling flexibility, which, in some cases, facilitated more open disclosure. No systematic differences in narrative depth were observed between modalities; however, analytic attention was paid to contextual features associated with each mode during interpretation.

The interview guide was pilot-tested with three eligible respiratory therapists who were not included in the final sample. Their feedback was used to refine question wording, improve clarity and flow, and ensure cultural appropriateness. The interview guide was conceptually informed by the study’s aims and designed to elicit experiential, relational, and interpretive accounts of burnout rather than checklist-style responses. Open-ended prompts encouraged participants to describe how burnout was experienced, understood, and navigated within their everyday clinical contexts, ensuring alignment between the research questions and the data generated.

Interviews lasted approximately 45–60 min and were audio-recorded following informed consent. All interviews were conducted in the participants’ native language, Arabic, to ensure culturally resonant and precise communication. Audio recordings were transcribed verbatim in Arabic, and selected excerpts were subsequently translated into English by bilingual members of the research team. Each translation was independently reviewed to ensure accuracy and consistency.

Translation was treated as an interpretive act rather than a purely technical process. The research team engaged reflexively with potential semantic shifts, particularly for emotionally laden or culturally specific expressions. Discrepancies in translated excerpts were discussed collaboratively to preserve contextual meaning and minimise interpretive loss.

The semi-structured interview guide ensured consistency while permitting exploration of new topics. It covered these areas:

- Outline of a typical workday and clinical duties;
- Personal signs and experiences of burnout;
- Views on organisational support and leadership;
- Primary workplace stressors and coping strategies;
- Considerations of job satisfaction and career plans;
- Suggestions for future RTs entering the profession.

Field notes were taken during and after interviews to document contextual observations, emotional tone, and preliminary analytic reflections. These notes were revisited during analysis to inform interpretation, contextualise participants’ verbal accounts, and support reflexive awareness throughout the analytic process.

### 2.4. Reflexivity

The interviews were conducted by academically trained respiratory therapists, positioning the researchers as professional insiders to the clinical context under study. This insider status facilitated rapport and contextual understanding, but also required critical, reflexive engagement to mitigate assumptions about shared professional norms, values, and experiences. Reflexivity was therefore treated as an ongoing analytic practice rather than a descriptive statement of researcher credentials.

The researchers’ professional proximity to respiratory therapy practice may have shaped interpretation through implicit normalisation of workplace stressors or shared understandings of burnout. To address this, reflexive questioning was used throughout the analysis to challenge taken-for-granted assumptions and to distinguish participants’ accounts from the researchers’ own professional experiences.

Reflexive practices were embedded within the analytic process through the use of reflexive memos, iterative team discussions, and deliberate examination of alternative interpretations during coding and theme development. Instances where researchers’ interpretations diverged were explicitly discussed to foreground participants’ meanings and enhance analytic transparency.

### 2.5. Data Analysis

An inductive thematic analysis was performed to identify recurrent patterns and explicit meanings, following the six-phase framework of Braun and Clarke [11]. This process was iterative, involving constant comparison across the entire. We looked for patterns that explained the underlying meaning behind the participants’ exhaustion, ensuring that each theme reached a level of analytic depth that reflected the interplay between institutional constraints and personal identity. To ensure accuracy and depth, all transcripts were reviewed against the original audio recordings. This familiarisation phase, conducted entirely in the original Arabic language to protect linguistic and cultural subtleties, allowed the transition from initial semantic codes to more complex latent themes that capture the lived experience.

NVivo 12 software assisted in data management for coding and audit-trail maintenance. This was combined with manual organisational methods, including hand-written reflexive memos and thematic mapping on paper, which allowed the researchers to maintain a close, tactile connection to the data. Two researchers collaboratively applied reflexive thematic analysis, engaging in ongoing discussion to develop and refine themes through shared interpretation.

The analysis progressed through three distinct levels of abstraction to ensure findings were grounded in the participants’ accounts. Initial open codes were first assigned to literal descriptions, such as ‘loss of passion’, ‘staff shortages’, ‘administrative disconnect’, and ‘seeking an alternative’. These were then grouped into intermediate categories based on conceptual similarity; for example, codes relating to lack of appreciation and poor management communication were clustered into the category of ‘Professional Marginalisation’. Finally, these categories were synthesised into the study’s overarching themes, such as ‘Institutional Entrapment’ and ‘Professional Invisibility’.

The research team critically reviewed the developed themes against the entire dataset to ensure they fully captured the complexity of the participants’ accounts. This involved actively looking for divergent or atypical experiences and weighing these against dominant narratives to ensure a balanced synthesis.

After finalising themes, illustrative quotes were translated into English by bilingual researchers and verified for accuracy and contextual integrity. This procedure follows established guidance that qualitative analysis should occur in the original language to avoid translational distortion [12]. Developed codes and themes were reviewed with the full research team to confirm they faithfully represented the dataset and to enhance the credibility of the analysis [11].

To ensure rigorous reporting, a 15-point checklist for good reflexive TA was followed in this study across each stage within the TA phases. Adhering to these guidelines ensured that the analytic process was documented with transparency and methodological integrity.

### 2.6. Ethical Considerations

Ethical approval was granted by the Research Ethics Committee at King Faisal University (Approval ID: KFU-REC-2025-SEP–ETHICS3588). All procedures complied with the Declaration of Helsinki and institutional guidelines for human subjects research.

Before participation, all respiratory therapists received a written information sheet that explained the purpose of the study, procedures, potential risks and benefits, and the voluntary nature of their participation. Recorded informed consent was obtained from each participant prior to the interview. Participants were assured that:

Participation was voluntary, and they could withdraw at any stage without providing a reason.

No personal or identifiable information was collected or published.

Audio recordings would be stored in encrypted, password-protected folders accessible solely to the research team and deleted after transcription and analysis.

Transcripts would be anonymised, and all reporting would protect participant confidentiality.

Participants were not required to have a formal clinical diagnosis of burnout to be eligible for inclusion. Burnout was explored as a self-experienced phenomenon rather than a clinically diagnosed condition. Participants were informed that discussions of work-related stress could be emotionally sensitive and were reminded that they could pause or stop the interview at any time. Researchers remained attentive to participants’ well-being during interviews. Ethical considerations also extended to the research team, given their professional proximity to the field. Reflexive practices and team discussions were used to manage potential emotional impacts associated with engaging with burnout narratives.

The study was conducted with utmost respect for participants’ privacy, autonomy, and psychological welfare.

## 3. Results

**Figure 1:**
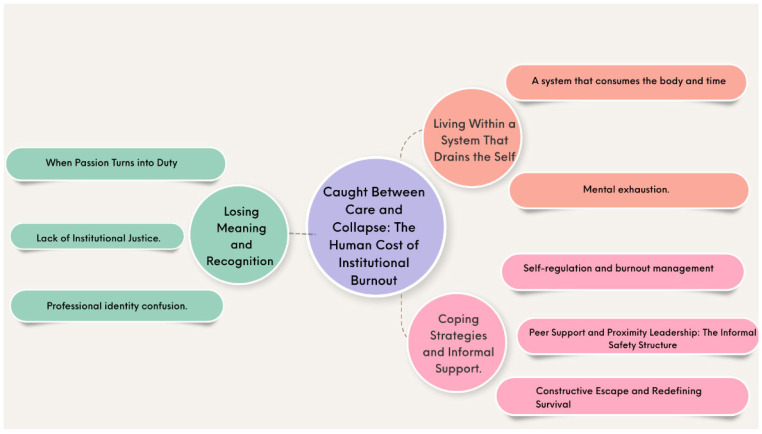
Schematic summary of themes and subthemes. This figure illustrates the overarching theme and three interrelated themes that emerged from the thematic analysis of respiratory therapists’ lived experiences with burnout. Each theme is presented with its corresponding subthemes, showing how systemic pressures, loss of meaning, and coping mechanisms collectively shape the burnout experience.

Interviews with RTs in Saudi Arabian government hospitals provided profound insights into burnout as a multifaceted and deeply personal ordeal. One central theme and three interrelated subthemes were developed to represent participants’ shared experiential patterns that recurred across accounts, reflecting common structural and organisational conditions rather than individual or demographic differences. These themes, summarised in Figure 1, reflect the perspectives of all participants.

### 3.1. Overarching Theme

#### Caught Between Care and Collapse: The Human Cost of Institutional Burnout

This overarching theme serves as the central narrative of this study. It encapsulates the emotional and ethical conflicts RTs face as they strive to uphold professional integrity and compassion within a system defined by persistent institutional pressures. This core tension is further explored through three subthemes: (1) the physical and emotional depletion caused by systemic demands, (2) the psychological impact of professional invisibility, and (3) the sense of institutional entrapment. Together, these themes demonstrate how structural and organisational conditions shape the lived reality of burnout for RTs.

Theme 1: Living Within a System that Drains the Self.

This theme serves as the foundational narrative of the conditions and pressures associated with burnout, illustrating how systemic and organisational factors such as chronic understaffing act as key contributors to RT burnout. This theme represents the first stage of the “Collapse,” where the therapist’s physical and temporal boundaries are overwhelmed by the needs of the institution.

Subtheme 1.1 A System that Consumes the Body and Time.

Severe staff shortages were identified by most as the primary factor contributing to burnout risk. They reported caring for as many as ten patients simultaneously, many of whom were critically ill, describing this ratio as both physically impossible and a source of deep moral distress. When a therapist is stretched across ten critically ill patients, care is reduced to a checklist of survival tasks rather than holistic therapy. P11 captured this sense of inadequacy, saying: “*Sometimes I’m alone with ten patients! I feel like I’m neglecting them all, but it’s really impossible for me to give them the attention they need.*” (P11). This highlights that the drain is not just physical; it is the psychological weight of a sense of powerlessness and knowing that they cannot provide the quality of care their professional ethics demands.

The 12-h shift structure was another major contributor to this depletion. This long hours schedule tethered RTs to the workplace, leaving little room for family or social engagement and evoking feelings of guilt and internal conflict between roles.

Participants described these shifts as a “daily battle” that inevitably encroaches on their private lives. P5 noted that such schedules “ *are very tiring and ruin your social life, especially if you have a family*.” This guilt is rooted in the conflict between their professional identity and their roles at home. “This tension was particularly strong among married participants, who described a heavier emotional burden when trying to balance these 12-h shifts with family responsibilities compared to their single colleagues. As P8 explained, the administrative and clinical load becomes so heavy that: “*The work becomes annoying rather than comfortable... if one or two employees are missing, it causes major problems for the whole system.*”

As the current structure feels unsustainable, participants identified specific systemic changes as the best path to restore professional balance. As P10 noted: “*I wish the shift would be changed to eight hours to make things easier.*”

The cumulative impact of these structural pressures, such as long hours, high patient loads, and emotional drain, led participants to describe stress not as simple fatigue but as a daily battle in which time becomes the enemy. As one participant reflected:

“*The pressure from the patient ratio and long hours changes care from a therapeutic practice into a series of tasks, with the sole goal being ‘get through the day*.’” (P1).

Although this study included participants from the ICU, ER, and general wards, their experience of ‘The Drain’ was consistent across all settings, regardless of their specific clinical assignment for the day.

Subtheme 1.2 Mental Exhaustion.

Interviews revealed a state of profound emotional exhaustion, where sustained physical tiredness transitioned into psychological depletion. This represents the point where the care aspect of the job begins to feel unsustainable. Many expressed that after years in high-stress environments like the ICU, their minds had become as weary as their bodies. This exhaustion transcended tiredness, becoming an emotional paralysis that eroded empathy, patience, and motivation and increased susceptibility to burnout. P9 shared a haunting account of this breaking point: *“I continued, continued, until I exploded at the wrong time... I didn’t want anyone to see me in that weakness*.

As the boundaries between their body and mind have blurred, the collapse often manifests in moments of raw, uncontrolled emotion. Continuous exposure to critically ill patients, combined with unrelenting demands and insufficient rest, left them unable to fully engage with their work or those around them. One participant admitted, “*I was doing my work while crying*” (P9), while another noted “*becoming impatient and without energy*” (P7).

For many, this state was more than fatigue. It represents the gradual disappearance of self within the system, where the clinician’s capacity to act as healer is constrained, leaving them as a reactive component of the hospital machinery. Compassion gave way to routine, as sustained exhaustion limited their ability to emotionally engage in care. This transformation was most poignantly summarised by P1, who described the end-stage of this process as becoming “*a machine that breathes for others but forgets to breathe for itself*”.

Theme 2. Losing Meaning and Recognition.

While Theme 1 describes the depletion of emotional and cognitive capacity under sustained workload, Theme 2 shifts focus to the erosion of meaning and professional value that emerged when such exhaustion was compounded by institutional neglect and lack of recognition. This stage marks a critical transition in the collapse, where therapists move from being physically depleted to experiencing a loss of purpose and professional fulfilment.

Subtheme 2.1: When Passion Turns into Duty.

Participants consistently reported that their work, once fueled by compassion, had been reduced under the weight of constant systemic pressure to mere obligation. This transition from a professional calling to a repetitive, emotionally detached routine represents a significant stage of the collapse, where internal resources are simply exhausted. As one therapist reflected on this shift, “*We’ve become just doing what’s required, without any motivation or passion*” (P5).

Their accounts revealed a transition from genuine motivation to emotional numbness. What drained their passion was not just a temporary state of fatigue, but it resulted in a permanent detachment from the clinical bedside. P 10 explained this point of no return, saying, “I’ve had enough of the bedside... if I think of leaving [this hospital], I won’t think of going to another hospital to be a bedside RT again. I’ve had enough”

This feeling of “ I’ve had enough” indicates that the feelings of burnout have evolved into withdrawal from the professional identity of a specialist. Similarly, P11 described how the depletion of energy leads to a decline in the quality of professional presence, where work is no longer a source of pride, but something performed “anyway”: “*I don’t want to reach a level where I have no energy and my work becomes ‘just anything’... it’s easier to just stay in a stable place with regular hours*.” This detachment signifies the moment where care is no longer the core objective. Instead, the clinician enters a survival mode, trading professional empathy for a psychological mechanism aimed at conserving their remaining emotional reserves.

Consequently, the overarching goal is no longer the patient’s recovery but surviving till the end of the shift.

In this sense, participants described a form of moral fatigue that reflects a key psychosocial dimension linked to burnout, where therapists continued to value patient care in theory but felt too “numb” to engage with it in practice.

While the loss of passion was common across accounts, the experience varied by seniority. Junior RTs often described a “reality shock,” marked by distress over feeling unable to provide adequate care due to ratios, as reflected in P11’s account. In contrast, senior RTs spoke of a deeper saturation that developed over time, as captured by P10 (nine years’ experience) who stated, “*I’ve had enough of the bedside… I have nothing more to give*.” This suggests that prolonged exposure to the same structural pressures shapes how professional disengagement emerges.

Subtheme 2.2: Organisational Injustice and Professional Misrecognition.

This disconnect between the frontline reality and managerial perception represents a secondary stage of the “Collapse,” where the struggle for care is silenced by the institution. *P*articipants consistently cited unfair treatment and poor leadership as key factors in their experiences of distress and burnout risk. They described a culture that valued compliance and public image over fairness and empathy. P7 stated, “*We demand simple rights, such as infection allowance, but the administration doesn’t care. They say, ‘This is your job*.’”.

Several RTs viewed leadership as symbolic rather than substantive, perceiving it as more invested in projecting an ideal image than confronting on-the-ground realities. As P4 noted, *“Management only wants to hear positive things,*” treating critique as a threat rather than a chance for improvement. Another participant confirmed, “*Management doesn’t want to hear about problems. They want us to say everything is fine, even if the situation isn’t perfect. The important thing is that the image looks good in front of them.”* (P5).

In some cases, participants were expected to achieve ideal performance despite inadequate human resources or limited equipment. P3 highlighted this contradiction*: “The manager talks about quality but doesn’t go out and see the real suffering in the department.”* Together, these narratives depict an organisation where appearance trumps compassion, and where fairness and open communication are replaced by silence and frustration.

This sense of injustice is compounded by the RT’s struggle within the interprofessional hierarchy. Participants reported that their specialised knowledge was often overlooked by other healthcare providers, limiting their scope for action and diminishing job satisfaction.

P11 explained, “*We spend our time justifying our work to nurses or doctors because they don’t understand the nature of our work*.” This indicates the conflict was not interpersonal but rooted in a misalignment of professional identity within an unbalanced system.

The constant effort to prove their importance drained therapists’ time and energy, diverting them from clinical duties to defending their professional relevance. As P9 observed, “*A major misunderstanding of the role…wasting the specialist’s time justifying protocols*”.

Taken together, these accounts suggest that organisational injustice operated at multiple levels through leadership practices that constrained voice and prioritised image, through expectations of “quality” without resources, and through interprofessional hierarchies that undermined recognition of the RT role.

In this context, participants framed their distress not as an individual failing, but a response to an unjust system that prioritises its external reputation over the professional dignity and practical needs of its staff.

However, variations in experience were observed in cases where leadership provided direct support rather than symbolic oversight. These contrasting accounts highlight how active engagement could buffer the sense of injustice. For instance, P8 described a manager who ‘comes with us during crowded times and helps’, fostering a sense of gratitude. The RTs valued this choice to act rather than offer excuses, yet they also recognised the leader’s limitations; they understood that while a supportive supervisor could ease the immediate burden, they remained unable to change the broader systemic constraints. This distinction between individual support and structural failure underscores that relational leadership, while vital, cannot fully compensate for the institutional ‘Collapse’”.

Theme 3. Coping Strategies and Informal Support.

This theme outlines the shared coping mechanisms and informal support systems RTs employed to navigate the emotional and systemic pressures of their roles. It consists of three subthemes that detail these strategies. These strategies did not resolve burnout risk but acted as a compensatory mechanism in the absence of organisational protection.

Subtheme 3.1: Self-Regulation and Burnout Management.

Many participants described active efforts to maintain psychological equilibrium through simple, effective practices. P5 stated, “*I try not to think about the issue as much as I can. I need to disconnect before I collapse*.”.

A common approach was taking short, preventive breaks to recover emotionally before reaching a breaking point. Many emphasised the importance of temporarily stepping away. As P11 said, “*I learned to take a break before I reach the breaking point, it helps me clear my mind and come back able to continue.*”.

Others highlighted the value of cultivating hobbies and interests outside work to restore balance and reinforce a non-professional identity. “*I try to keep myself busy with something I love outside work so I can feel I have a life beyond the job*”, explained P5.

Some participants also used brief mindfulness practices during shifts, such as breathing exercises or silent reflection, to manage acute stress. P9 noted, *“These few minutes saved me from exploding*”.

These actions were not viewed as luxuries but as essential self-regulation practices that allowed them to remain effective in a demanding environment. Collectively, they reflected an increasing self-awareness and psychological adaptability, an acknowledgement that when institutions fail to protect their staff, personal strategies become the only defence against collapse.

Subtheme 3.2: Peer Support and Proximity Leadership: The Informal Safety Structure.

Interviews revealed that peer relationships provided more support than any formal institutional offering. P10 noted, “*Talking to colleagues comforts me more than anything*. *We try to laugh even in the middle of a crowd.*” This highlights the power of emotional solidarity, where friendship forms a psychological network that shares the burden of stress, preventing individual breakdowns. Importantly, reliance on peer solidarity and proximity leadership emerged in place of, rather than in addition to, formal organisational support, underscoring participants’ mistrust of institutional mental health structures.

The role of compassionate, hands-on leadership in reducing burnout was also emphasised. P8 shared, “My manager comes with us during crowded times and helps. This helps us a lot.” Similarly, P4 noted, “*As a leader, I try to demand employees’ rights. I feel it is my duty to protect them*.” These accounts demonstrate that compassionate and engaged leadership functions as a protective buffer against institutional rigidity. While such leadership cannot eliminate systemic pressures, it can slow their emotional impact and help rebuild the trust employees have lost in the system.

Conversely, formal mental health services were viewed as largely ineffective. Despite their availability in some hospitals, participants often avoided them due to stigma and fear of being seen as weak. As P9 mentioned, “*There’s a mental health clinic in the hospital, but no one goes. They’re afraid someone might find out or think they broke down*.”.

This reluctance signals a lack of trust in institutional support systems, which are often perceived as symbolic rather than genuinely safe. Consequently, participant resilience was forged not through formal structures but through informal peer networks that served as the true psychological shield against burnout.

Subtheme 3.3: Constructive Escape and Redefining Survival.

When daily coping mechanisms proved insufficient, therapists sought deeper ways to recalibrate their careers. Some described pursuing further education or transitioning to less demanding roles as an act of survival, not abandonment. P11 remarked, *“I am completing a study to escape from working with patients*,” while another participant expressed a desire to shift to a more regular working hours: “*I am thinking of switching to a PFT clinic; it’s more comfortable for me*.” (P10).

These decisions were not acts of escape but deliberate strategies to reframe the professional relationship. Participants explained that moving from high-pressure to calmer environments or pursuing further education helped them regain control over their work lives.

However, many acknowledged that leaving was not a feasible option. Financial obligations, family duties, and limited alternatives bound them to their positions. As P5 observed, “*Friends in other hospitals complain about similar or even harsher conditions*,” suggesting a lateral move would not bring relief. Another participant echoed this constraint: “*Financial debt is the reason I stay; I have no other choice*” (P2).

Despite this, several participants expressed a moral and emotional commitment that superseded material concerns. P4 reflected, “*Despite the financial incentives, I am done with this. The quality of life matters more than money.”* P8 added an ethical perspective*:* “*I love my work and feel a responsibility towards the people of my region, but the system consumes us.*”.

This paradox illustrates that remaining in the profession is not always an act of surrender. For some, endurance became a form of moral resistance, an implicit act of integrity through which therapists upheld the human value of care, even when institutional systems failed to protect them.

Taken together, these coping strategies illustrate how RTs managed ongoing exposure to burnout risk factors rather than escaping them, reinforcing that individual resilience functioned as a temporary buffer against institutional failure, not a substitute for structural prevention.

## 4. Discussion

This qualitative study investigated the lived experience of burnout among RTs in Saudi Arabia, contributing to a significant gap in the literature. The findings reveal that burnout is not a series of isolated symptoms but a progressive trajectory of professional erosion. Theme 1 (The Drain) acts as the structural foundation, where resource scarcity initiates physical strain. This facilitates the transition into Theme 2 (The Collapse), where the experience shifts from physical tiredness to a crisis of professional identity and organisational injustice. Finally, Theme 3 (Coping) represents the informal mechanisms used to navigate this unresolved tension. Consistent with prior quantitative research [6,7], participants reported severe exhaustion driven by persistent understaffing, extended shifts, and excessive workloads. This “Drain” can be analysed through the Job Demand–Control (JDC) model, where the combination of high psychological demands (e.g., 1:10 patient ratios) and low decision-making latitude creates a high-strain environment [13]. While several Western studies link this level of burnout directly to turnover, our participants demonstrated persistence despite profound exhaustion [14]. This endurance signifies a distinct moral dimension, sustained professional duty and responsibility toward patients that override personal distress. Importantly, this pattern is not unique to Saudi Arabia. For instance, a qualitative study from the European healthcare system similarly reports that peer solidarity and moral obligations keep professionals in the occupation despite systemic strain [9]. Furthermore, our results suggest that this persistence is heavily influenced by the lack of specialised job alternatives within a feasible radius. This interpretation aligns with occupational psychology research showing that limited job alternatives and perceived career immobility contribute to continued employment even in undesirable conditions [15]. Consequently, lower turnover in this context may mask a state of “silent endurance” where RTs remain in roles that negatively impact their health because no viable exit exists.

Participants also emphasised the emotional erosion stemming from a loss of meaning and recognition. Many RTs felt invisible within their institutions, a finding that resonates with studies on Canadian and U.S. therapists [16,17]. This experience is effectively explained by the Effort–Reward Imbalance (ERI) model, where high effort (emotional endurance) is not met with extrinsic rewards such as institutional fairness or professional misrecognition [18]. Similar to other Saudi allied health professionals, participants reported that open communication and leadership acknowledgement were rare, while administration often prioritised institutional reputation [19]. These findings corroborate evidence that poor leadership support increases depersonalisation and suggest that the hierarchical culture in Saudi healthcare, which discourages questioning authority, deepens emotional withdrawal [10]. The “Collapse” identified in Theme 2 represents a state of moral injury, where practitioners are constrained by structural limitations from acting according to their professional values.

The third theme, Coping Strategies and Informal Support, reveals how RTs navigated unsupportive environments. Many depended on informal peer solidarity, humour, and self-distraction to manage stress, consistent with patterns seen among nurses [19] and North American RTs [16]. A significant finding was the widespread distrust of institutional mental health services. Participants avoided workplace counselling due to stigma and confidentiality concerns, a pattern also reported among Saudi medical residents trapped in a burnout stigma cycle that deters help-seeking [20].

Thus, RTs cultivated informal support networks, relying on colleagues’ understanding and proximity-based leadership, in which supervisors offered direct, hands-on assistance. This aligns with team-based coping used by Canadian RTs to handle workplace stress [16]. Overall, these findings indicate that in rigid, resource-scarce environments, informal strategies partially offset inadequate institutional support, though systemic organisational change remains imperative.

Our study thus broadens the understanding of burnout among RTs in the Middle East. It reveals that, beyond the well-known dimensions of burnout as described by Maslach and Jackson [21], burnout is also mediated by moral conflict, mistrust, and cultural silence. The reluctance to seek internal support, persistence in the absence of replacements, and dependence on informal peer networks represent distinctive expressions of burnout within this healthcare culture.

In summary, moving beyond descriptive accounts allows us to see burnout as a theorised outcome of structural strain and reciprocity deficits. While the triggers for burnout among Saudi RTs are universal, retention is mediated by a mix of moral defiance and localised economic constraints.

## 5. Implications for Clinical Practice and Policy

The study’s findings carry important implications for practice and policy. Institutionally, addressing the systemic roots of burnout suggests a need for comprehensive reforms that prioritise workforce well-being. The structural ‘Drain’ identified in Theme 1 implies that chronic understaffing, long shifts, and excessive workloads should be addressed through equitable staffing models, flexible scheduling, and workload redistribution to ensure clinical endurance. Providing sufficient rest and opportunities for psychological recovery may mitigate the pervasive exhaustion reported by therapists.

Leadership conduct was a crucial factor in emotional well-being. The disconnect between managerial perception and frontline reality was strongly linked to depersonalisation and moral fatigue. Therefore, our data suggests that hospital leaders should consider training in supportive, participatory styles that emphasise transparency, empathy, and acknowledgement of staff efforts to correct the identified deficits. Leadership development integrating emotional intelligence and proximity principles could enhance psychological safety and foster a stronger sense of belonging.

Furthermore, the widespread avoidance of workplace counselling due to stigma suggests that institutions should aim to normalise mental health access via confidential, culturally sensitive programs. Integrating peer support and structured debriefing after stressful clinical events could further bolster resilience and collective coping.

Nationally, our findings suggest that recognising RTs as a strategic priority is essential for Saudi Arabia’s healthcare transformation. Including RTs in decision-making committees, professional development, and workforce planning may ensure that burnout prevention strategies are both evidence-based and contextually appropriate. Such measures could improve retention, reinforce professional identity, and secure the long-term sustainability of this essential workforce.

## 6. Strengths and Limitations

This study offers the first qualitative account of burnout among Saudi RTs, capturing lived experiences that surveys cannot. The use of in-depth, language-sensitive Arabic interviews enabled participants to express culturally nuanced meanings with authenticity. Reflexive analysis and peer debriefing further strengthened the credibility and transparency of the findings.

However, as with all qualitative research, the results are context-specific and not universally generalisable. Participants were recruited mainly from government hospitals, whose culture may differ from that of private institutions. Social desirability bias may have influenced discussions of leadership or coping. Finally, despite rigorous translation, subtle linguistic nuances may have been partially lost in the English translation of quotations.

## 7. Conclusions

This study provides a detailed, experience-driven analysis of burnout among Saudi RTs, addressing a critical literature gap. By exploring the lived experience of being “Caught Between Care and Collapse,” this study reveals that systemic strain, inadequate recognition, and institutional mistrust foster emotional exhaustion and moral conflict. The findings demonstrate that therapists are suspended in a state of tension, which is driven by a profound “Care” for their patients, yet pushed toward professional “Collapse” by structural neglect. This study’s original contribution lies in synthesising the JDC and ERI models to demonstrate how many therapists reported feeling structurally trapped, remaining in their roles out of a sense of duty and limited options rather than through active engagement. Despite organisational neglect, they depended on peer support and personal resilience to maintain patient care. We conclude that burnout in this context is not a failure of individual resilience, but a theorised outcome of structural strain and reciprocity deficits. These findings highlight the analytical need for culturally informed, system-level interventions to mitigate burnout and protect the well-being of this vital healthcare workforce.

## Figures and Tables

**Table 1:** Characteristics of participants.

Psudenomuos ID	Gender	Location	Years of Experience
P1	Male	Western region	10
P2	Male	Western region	10
P3	Male	Southern region	5
P4	Male	Northern region	9
P5	Female	Western region	5
P6	Male	Eastern Region	9
P7	Female	Western region	5
P8	Male	Central region	22
P9	Female	Western region	7
P10	Female	Eastern region	9
P11	Female	Western region	3.5

## Data Availability

The data presented in this study are available on request from the corresponding author. The data are not publicly available due to privacy and ethical restrictions.

## References

[1] Maslach C., Schaufeli W.B., Leiter M.P. (2001). Job burnout. Annu. Rev. Psychol..

[2] Guttormson J.L., Calkins K., McAndrew N., Fitzgerald J., Losurdo H., Loonsfoot D. (2022). Critical care nurse burnout, moral distress, and mental health during the COVID-19 pandemic: A United States survey. Heart Lung.

[3] Quesada-Puga C., Izquierdo-Espin F.J., Membrive-Jimenez M.J., Aguayo-Estremera R., Cañadas-De La Fuente G.A., Romero-Bejar J.L., Gomez-Urquiza J.L. (2024). Job satisfaction and burnout syndrome among intensive-care unit nurses: A systematic review and meta-analysis. Intensive Crit. Care Nurs..

[4] Voultsos P., Koungali M., Psaroulis K., Boutou A.K. (2020). Burnout syndrome and its association with anxiety and fear of medical errors among intensive care unit physicians: A cross-sectional study. Anaesth. Intensive Care.

[5] Li L.Z., Yang P., Singer S.J., Pfeffer J., Mathur M.B., Shanafelt T. (2024). Nurse burnout and patient safety, satisfaction, and quality of care: A systematic review and meta-analysis. JAMA Netw. Open.

[6] Algarni S.S., Algihab A.A., Bin Dahmash H.A., Alomaireni A.S., Alzahrani R.A., Alruwaili A.H., Alotaibi N.N., AbuNurah H.Y., Alotaibi T.F., Ismaeil T. (2022). Burnout Among Respiratory Therapists in a tertiary hospital in Saudi Arabia. Respir. Care.

[7] Siraj R.A., Alhaykan A.E., Alrajeh A.M., Aldhahir A.M., Alqahtani J.S., Bakhadlq S., Alghamdi S.M., Alqarni A.A., Alqarni M.M., Alanazi T.M. (2023). Burnout, resilience, supervisory support, and quitting intention among healthcare professionals in Saudi Arabia: A national cross-sectional survey. Int. J. Environ. Res. Public Health.

[8] Krishna G.G., Harbli N.M.A., Krishnan N., Ghundul L.B., Aldhahri R., Aldossary A.B., Margalani A.O., Almeshari M., Alwadeai K.S., Alshehri R.A. (2025). Burnout among ICU and non-ICU healthcare professionals in Saudi Arabia: A comparative cross-sectional analysis. J. Multidiscip. Healthc..

[9] Colbenson G.A., Ridgeway J.L., Benzo R.P., Kelm D.J. (2021). Examining burnout in interprofessional intensive care unit clinicians using qualitative analysis. Am. J. Crit. Care.

[10] Omar A.S., Hanoura S., Labib A., Kaddoura R., Rahhal A., Al-Zubi M.M., Galvez R.D., Shiju S., Al Jonidi M.J., Ragab H. (2022). Burnout among respiratory therapists and perception of leadership: A cross sectional survey over eight intensive care units. J. Intensive Care Med..

[11] Braun V., Clarke V. (2021). Thematic Analysis: A Practical Guide.

[12] Van Nes F., Abma T., Jonsson H., Deeg D. (2010). Language differences in qualitative research: Is meaning lost in translation?. Eur. J. Ageing.

[13] Kain J., Jex S. (2010). Karasek’s (1979) job demands-control model: A summary of current issues and recommendations for future research. New Developments in Theoretical and Conceptual Approaches to Job Stress.

[14] Cerela-Boltunova O., Millere I., Nagle E. (2025). Moral Distress, Professional Burnout, and Potential Staff Turnover in Intensive Care Nursing Practice in Latvia—Phase 1. Int. J. Environ. Res. Public Health.

[15] Fahlén G., Goine H., Edlund C., Arrelöv B., Knutsson A., Peter R. (2009). Effort-reward imbalance, “locked in” at work, and long-term sick leave. Int. Arch. Occup. Environ. Health.

[16] Saragosa M., Goraya F., Nowrouzi-Kia B., Gohar B. (2024). A qualitative study examining stressors among Respiratory Therapists in Ontario amidst the COVID-19 pandemic. PLoS ONE.

[17] Strickland S.L., Roberts K.J., Smith B.J., Hoerr C.A., Burr K.L., Hinkson C.R., Rehder K.J., Miller A.G. (2022). Burnout among respiratory therapists amid the COVID-19 pandemic. Respir. Care.

[18] Siegrist J., Fink G. (2016). Chapter 9—Effort-Reward Imbalance Model. Stress: Concepts, Cognition, Emotion, and Behavior.

[19] Jaber M.J., Bindahmsh A.A., Baker O.G., Alaqlan A., Almotairi S.M., Elmohandis Z.E., Qasem M.N., AlTmaizy H.M., du Preez S.E., Alrafidi R.A. (2025). Burnout combating strategies, triggers, implications, and self-coping mechanisms among nurses working in Saudi Arabia: A multicenter, mixed methods study. BMC Nurs..

[20] Alwatban L., Alageel M.S., Alshehri L.A., Alfehaid N.S., Albahlal R.A., Almazrou N.H., Almubarak R. (2024). The Stigma of Burnout Impeding Formal Help: A Qualitative Study Exploring Residents’ Experiences During Training. Adv. Med. Educ. Pract..

[21] Maslach C., Jackson S. (1981). The measurement of experiencedburnout. J. Organ. Behav..

